# Regulation of Murine Ovarian Epithelial Carcinoma by Vaccination against the Cytoplasmic Domain of Anti-Müllerian Hormone Receptor II

**DOI:** 10.1155/2015/630287

**Published:** 2015-11-05

**Authors:** Cagri Sakalar, Suparna Mazumder, Justin M. Johnson, Cengiz Z. Altuntas, Ritika Jaini, Robert Aguilar, Sathyamangla V. Naga Prasad, Denise C. Connolly, Vincent K. Tuohy

**Affiliations:** ^1^Department of Immunology, Lerner Research Institute, Cleveland Clinic, Cleveland, OH, USA; ^2^School of Medicine, Department of Medical Biology, Genome and Stem Cell Research Center, Erciyes University, 38039 Kayseri, Turkey; ^3^Department of Biology, Cleveland State University, Cleveland, OH, USA; ^4^North American University, Texas Institute of Biotechnology, Education, and Research, 10555 Stella Link Road, No. 102, Houston, TX 77025, USA; ^5^Western Reserve Academy, Hudson, OH, USA; ^6^Department of Molecular Cardiology, Lerner Research Institute, Cleveland Clinic, Cleveland, OH, USA; ^7^Developmental Therapeutics Program, Fox Chase Cancer Center, Philadelphia, PA, USA; ^8^Department of Molecular Medicine, Cleveland Clinic Lerner College of Medicine, Case Western Reserve University, Cleveland, OH, USA

## Abstract

Anti-Müllerian hormone receptor, type II (AMHR2), is a differentiation protein expressed in 90% of primary epithelial ovarian carcinomas (EOCs), the most deadly gynecologic malignancy. We propose that AMHR2 may serve as a useful target for vaccination against EOC. To this end, we generated the recombinant 399-amino acid cytoplasmic domain of mouse AMHR2 (AMHR2-CD) and tested its efficacy as a vaccine target in inhibiting growth of the ID8 transplantable EOC cell line in C57BL/6 mice and in preventing growth of autochthonous EOCs that occur spontaneously in transgenic mice. We found that AMHR2-CD immunization of C57BL/6 females induced a prominent antigen-specific proinflammatory CD4+ T cell response that resulted in a mild transient autoimmune oophoritis that resolved rapidly with no detectable lingering adverse effects on ovarian function. AMHR2-CD vaccination significantly inhibited ID8 tumor growth when administered either prophylactically or therapeutically, and protection against EOC growth was passively transferred into naive recipients with AMHR2-CD-primed CD4+ T cells but not with primed B cells. In addition, prophylactic AMHR2-CD vaccination of TgMISIIR-TAg transgenic mice significantly inhibited growth of autochthonous EOCs and provided a 41.7% increase in mean overall survival. We conclude that AMHR2-CD vaccination provides effective immunotherapy of EOC with relatively benign autoimmune complications.

## 1. Introduction

Epithelial ovarian cancer (EOC) is the leading cause of death from gynecologic malignancies in the United States [1, 2]. Approximately 60% of ovarian cancers are diagnosed at late stages, and although initial responses to the current standard of care are high, most patients have disease recurrence resulting in a five-year overall survival (OS) rate slightly over 45% [2, 3]. The high rate of ovarian cancer recurrence and the low five-year survival rate indicate the urgency for more effective ways to control this disease.

Induction of ovarian tumor immunity through vaccination is a promising approach and finds support from the increased OS observed in patients whose ovarian tumors are infiltrated by T cells [4]. Several therapeutic ovarian cancer vaccine strategies have been employed using whole tumor homogenate strategies as well as approaches involving targeted immunity against tumor associated antigens (TAA) overexpressed in ovarian malignancies including human epidermal growth factor receptor 2 (HER2), cancer-testis antigen 1 (CTAG1B or NY-ESO-1), or cancer antigen 25 (CA-125) [5]. Thus far, targeted immunity against these non-ovarian-specific TAA has provided modest therapeutic results [6–8].

In contrast, vaccination against tissue-specific differentiation antigens has not been fully exploited for providing ovarian cancer therapy despite the ability of such targeted vaccinations to increase OS in melanoma and prostate cancer patients [9–11]. Thus, vaccination against differentiation proteins expressed at immunogenic levels predominantly in the tissue from which the tumor is derived may provide effective immunotherapy against established tumors and at the same time substantially lower risk of inducing systemic autoimmune inflammatory complications.

We selected mouse anti-Müllerian hormone receptor II (AMHR2, GenBank ID: 110542) as our target differentiation protein for ovarian cancer vaccination because its full-length expression in normal human tissues is confined to the ovary and because it is also expressed in the vast majority of human EOCs including 90% of primary EOCs, 78% of borderline malignancies, 77–86% of non-EOC ovarian tumors, and 56% of malignant ascites from grades III-IV ovarian cancers [12–14].

AMHR2 is a serine/threonine kinase receptor homologous to type II receptors of the transforming growth factor beta (TGF*β*) family [15]. The human* AMHR2* gene contains 11 exons with seven known alternatively spliced variants producing three known coded proteins, one additional variant with protein coding features, and three noncoding transcripts with no open reading frames [16, 17]. In adult women, the longest human protein coding transcript for a 573-amino acid long protein is normally expressed only in the ovary and comprises a 127-amino acid extracellular domain, a 26-amino acid transmembrane domain, and a 403-amino acid cytoplasmic domain [16, 17]. AMHR2 signaling causes regression of the Müllerian ducts during male development and regulates oocyte development and follicle production in adult females thereby providing substantial control of ovarian reserve and fertility [15, 18–20].

Based on its expression in 90% of primary human EOCs as well as on its relatively confined distribution in normal human tissues, we hypothesized that AMHR2 vaccination would provide effective immunotherapy against EOC without producing extensive autoimmune complications. We tested our hypothesis using both transplantable and autochthonous mouse models for EOC. Mouse ID8 cells, derived from repeated* in vitro* passage of mouse ovarian surface epithelial cells (MOSEC), form EOCs when inoculated into C57BL/6 mice [21]. TgMISIIR-TAg transgenic mice develop bilateral autochthonous EOCs due to expression of the simian virus 40 large T antigen (SV40-TAg) under control of the* AMHR2* promoter [22].

All efforts to generate a full-length AMHR2 protein proved futile due to extensive toxicity in all expression systems tested. We resolved this toxicity problem by generating a recombinant mouse AMHR2 protein consisting of a 399-amino acid sequence of the cytoplasmic domain (AMHR2-CD) and found that immunization with this fragment resulted in a prominent proinflammatory T cell response accompanied by extremely high IgG antibody titers. Vaccination with AMHR2-CD provided highly significant T cell-mediated prophylaxis and therapy against ID8 EOC and mediated significant prophylaxis against the development of autochthonous EOCs in TgMISIIR-TAg transgenic mice. Moreover, the protection against tumor growth was accompanied by a rather benign autoimmune phenotype. Our data indicate that targeted vaccination against AMHR2-CD provides relatively safe and highly effective therapy against EOC.

## 2. Materials and Methods

### 2.1. Generation of Recombinant Mouse AMHR2-CD

**Figure 1:**
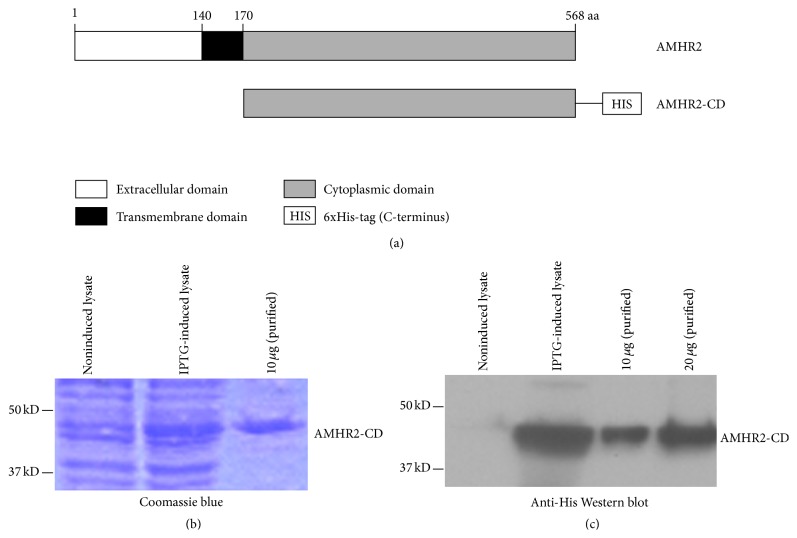
*Generation of recombinant mouse AMHR2-CD*. (a) Schematic representation of full-length AMHR2 showing the extracellular, transmembrane, and cytoplasmic domains with a C-terminal 6xHis-tagged AMHR2-CD variant. (b) Expression of AMHR2-CD in noninduced, IPTG-induced, and Ni-NTA affinity purified AMHR2-CD shown on an SDS-PAGE gel stained with Coomassie blue. (c) Anti-His Western blot of SDS-PAGE gel showing expression of AMHR2-CD in noninduced, IPTG-induced, and two doses of Ni-NTA affinity purified AMHR2-CD.

mRNA was extracted from ovaries of 8-week-old female C57BL/6 mice. Primer pairs designed to amplify the AMHR2 sequence 170–568 were used to generate the entire 399-amino acid cytoplasmic domain of mouse AMHR2 by RT-PCR [23]. To optimize protein folding and enhance overall yield, substitutions for native codon sequences were made (Dapcel, Cleveland, OH), and the optimized cDNA was inserted into the NdeI-Bam HI site of pET-3a (Novagen, Darmstadt, Germany) thereby providing a C-terminal 6xHis-tagged recombinant protein (Figure 1(a)). Plasmids containing these inserts were transformed in* E. coli* (Lucigen, Middleton, WI). High level expression colonies were selected following induction with Isopropyl *β*-D-1-thiogalactopyranoside (IPTG, Amresco, Solon, OH) and were sequenced for confirming proper orientation and alignment. The 6xHis-tagged AMHR2-CD was purified under denaturing conditions using nickel-nitrilotriacetic acid (Ni-NTA) affinity chromatography (Qiagen Sciences, Germantown, MD). The purified AMHR2-CD was electrophoresed on denaturing SDS-PAGE gels (Bio-Rad, Hercules, CA) and blotted onto immunoblot PVDF membrane (Bio-Rad). Immune detection of AMHR2-CD was performed using the enhanced chemiluminescence system (Amersham Biosciences, Piscataway, NJ) with HRP-conjugated His antibody (Qiagen). Prior to use, the 6xHis-tagged AMHR2-CD was purified by reverse phase HPLC to yield endotoxin-free protein [24]. Levels of endotoxin were <0.05 endotoxin units (<5 pg) per mg of recombinant protein.

### 2.2. Mice and Immunization

Female C57BL/6 mice served as recipients of ID8 tumors. They were obtained commercially (Jackson Laboratory, Bar Harbor, ME) at six weeks of age and immunized at 7–10 weeks of age by subcutaneous injection in the abdominal flanks with 100 *μ*g of recombinant mouse AMHR2-CD in 200 *μ*L of an emulsion of equal volumes of water and complete Freund's adjuvant (CFA, Difco, Detroit, MI) containing 400 *μ*g of* Mycobacteria tuberculosis*. TgMlSIIR-TAg (DR26 line) transgenic mice (provided by DDC) were maintained by breeding male TgMISIIR-TAg (H-2^b^) mice to wild-type syngeneic C57BL/6 females (Jackson Laboratory). TgMlSIIR-TAg mice were immunized at 6-7 weeks of age with 100 *μ*g of recombinant mouse AMHR2-CD in CFA as described above. To determine fertility phenotypes, age-matched test and control vaccinated C57BL/6 female mice were mated with the same C57BL/6 males. All protocols were preapproved by Cleveland Clinic's Institutional Animal Care and Use Committee.

### 2.3. Tumor Inoculation and Measurement

The ID8 EOC cell line was generously provided by Dr. Kathy Roby (University of Kansas Medical Center, Kansas City, KS). ID8 cells were cultured in 75 or 225 cm^2^ tissue culture flasks (BD Biosciences, Franklin Lakes, NJ) in DMEM (Mediatech Cellgro, Manassas, VA) containing 4% fetal bovine serum (Thermo Scientific Hyclone, Logan, UT), 1% penicillin/streptomycin (Invitrogen, Carlsbad, CA), and insulin-transferrin-sodium selenite media supplement (Sigma-Aldrich, St. Louis, MO) until the cells became 70–80 % confluent. Cells were harvested by trypsinization and washed twice with PBS. Female C57BL/6 mice were inoculated subcutaneously in the left dorsal flank with 5 × 10^6^ ID8 cells. Growth of ID8 tumors was assessed regularly by using a Vernier caliper to measure length × width. Tumor growth endpoint was determined by a measurement in any direction of 17 mm.

### 2.4. *In Vivo* Imaging and Measurement of Autochthonous Ovarian Tumors

Bilateral ovarian tumor growth in female transgenic mice was measured monthly by ultrasound using the Vevo 770 high-resolution* in vivo* microimaging system for small animals (VisualSonics, Toronto, Canada). Real-time imaging of the abdomen was performed using the RMV704 low frequency probe/scan head and aqueous conductive gel after removing hair from the abdominal region. Anesthesia for immobilization was administered using a nose cone with continuous flow of 1-2% vol/vol isoflurane during the image acquisition period lasting less than 30 minutes, and oxygen supply was continuously maintained. The probe/scan head was moved over the abdominal area very gently after applying aqueous conductive gel. Measurements and calculation of tumor area were performed using the Vevo software B-Mode measurement tool allowing for a 2D assessment of ovarian tumor size* in vivo* with the polygon region of interest setting (VisualSonics). Measurement of solid tumor size by B-mode sonography has been shown to correlate well with histopathologic measurement [25].

### 2.5. RT-PCR

Tissues were excised and stored frozen in RNA-Later (Life Technologies, Grand Island, NY). RNA was extracted by tissue homogenization in TRIZOL reagent (Invitrogen), and cDNA was generated from bulk RNA using Superscript III (Invitrogen). Gene expression was quantified by qRT-PCR using SYBR Green PCR mix (Applied Biosystems, Carlsbad, CA) with gene-specific primers (Table 1). Relative gene expression was assessed by normalization of each test gene expression level to *β*-actin expression levels in each individual tissue. Gene expression was determined by conventional RT-PCR using AMHR2-specific and *β*-actin-specific primers (Table 1). After amplification through 30 cycles, PCR products were separated on agarose gels (2% in 1 TBE buffer) and visualized under ultraviolet light after staining with ethidium bromide. Transgene expression in offspring of TgMlSIIR-TAg mice was determined by PCR amplification of a 773 bp fragment of SV40-TAg using primer pairs as previously described [22] (Table 1).

### 2.6. Flow Cytometry Analysis of Tumor Infiltrating Lymphocytes (TILs)

TILs were isolated from ID8 tumors by digestion of minced tumor for 30 minutes at 37°C in HBSS containing 50 KU of DNase I (Sigma-Aldrich) and 0.2 mg/mL collagenase II (Life Technologies) followed by discontinuous gradient centrifugation. The partially purified TILs were treated with Fc*γ* III/II receptor antibody (BD Biosciences) in PBS containing 0.5% BSA and 0.05% sodium azide and double-stained with FITC-conjugated anti-mouse CD3 and either PE-conjugated anti-mouse CD4 or PE-conjugated anti-mouse CD8 (BD Biosciences). The CD3+ T cell population was gated and analyzed for percentages of CD4+ and CD8+ T cells. Data collected on 30,000 total events were analyzed using FlowJo software (BD Biosciences).

### 2.7. Passive Transfer of Tumor Immunity

Ten days after immunization of female C57BL/6 mice with AMHR2-CD or ovalbumin (OVA, Sigma-Aldrich) as an irrelevant control immunogen, LNCs at 5 × 10^6^ cells/mL were activated* in vitro* with 20 *µ*g/mL of immunogen in the presence of IL-12 (10 ng/mL) and IL-18 (10 ng/mL; Peprotech, Rocky Hill, NJ) in 24-well flat-bottom Falcon plates (BD Biosciences) in a total volume of 2.0 mL/well in DMEM supplemented as described above. After 3 days of restimulation, 2 × 10^7^ activated whole LNCs were injected intraperitoneally into sublethally.

*γ*-irradiated (5 Gy) naive female recipients. In another protocol, C57BL/6 female mice were immunized with either AMHR2-CD or OVA, and four weeks later, three groups of cells were injected intraperitoneally into sublethally *γ*-irradiated (5 Gy) naive female recipients including 7.5 × 10^7^ whole splenocytes reactivated with immunogen, IL-12, and IL-18 as described above, 2 × 10^7^ similarly reactivated CD4+ T cells purified from whole splenocytes by magnetic bead separation, and 2 × 10^7^ nonreactivated B220+ B cells also purified from whole splenocytes by magnetic bead separation. In all cases, hosts were inoculated subcutaneously on the day after cell transfer with 5 × 10^6^ ID8 cells, and tumor growth was assessed regularly as described above. Purities of enriched cells were determined by flow cytometry analysis using CellQuest software (BD Biosciences) and were consistently found to be >90%.

### 2.8. Immunologic Assays

T cell proliferation, ELISA assays for cytokine production, and immunohistochemical analysis were performed as previously described [26] and are detailed in supplemental material.

### 2.9. Biostatistical Analysis

Differences between mRNA expression levels and mean tumor weights were compared using Student's* t*-test. Differences between tumor growth curves were compared by unweighted one-way ANOVA, and differences in mouse survival curves were compared using the log-rank test.

## 3. Results

### 3.1. Generation of Recombinant Mouse AMHR2-CD

All attempts to express the full-length sequence of mouse AMHR2 in any expression system consistently caused cytotoxicity and failure to produce high expression colonies. To overcome this persistent cytotoxic effect, we expressed the longest hydrophilic domain of mouse AMHR2 consisting of the 170–568 sequence comprising the 399 amino acids of the entire cytoplasmic domain (Figure 1(a)). The Ni-NTA affinity purified C-terminal 6xHis-tagged protein migrated as a ~44 kD protein as determined by Coomassie blue staining of an SDS-PAGE gel (Figure 1(b)) and by Western blot immunostaining using HRP-conjugated His-specific antibody (Figure 1(c)).

### 3.2. Immunogenicity of AMHR2-CD

**Figure 2:**
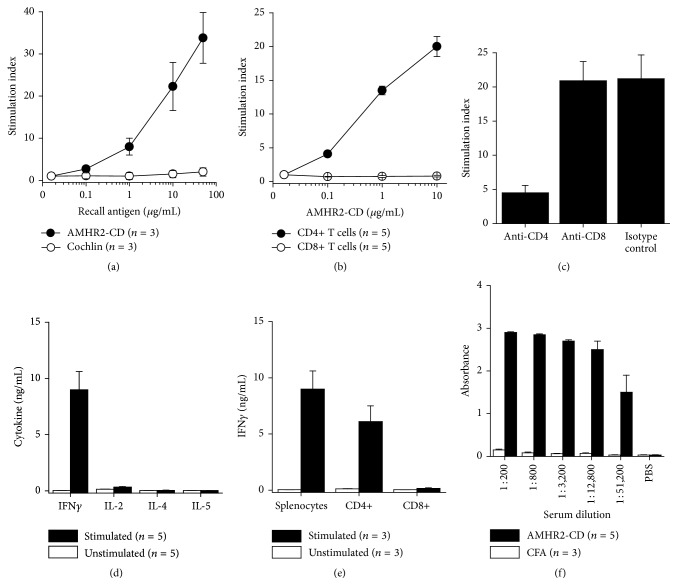
*Immunogenicity of AMHR2-CD*. Female C57BL/6 mice were immunized with AMHR2-CD in CFA, and LNC or splenocytes were cultured* in vitro* for assessment of proliferation and cytokine production. (a) Ten-day primed LNC showed marked antigen-specific recall proliferative responses to AMHR2-CD over several logs of antigen concentration. (b) The response to AMHR2-CD was elicited by CD4+ T cells but not by CD8+ T cells purified by magnetic bead separation. (c) Proliferative responses to AMHR2-CD were markedly inhibited in the presence of CD4 antibody but not in the presence of CD8 or isotype control antibodies. (d) Four weeks after immunization, splenocytes were reactivated with immunogen and ELISA analysis of 72-hour culture supernatants showed that recall responses to AMHR2-CD involved a proinflammatory phenotype with elevated production of IFN*γ* and minimal production of IL-2, IL-4, and IL-5. (e) Splenocyte production of IFN*γ* was elicited from purified CD4+ T cells but not from purified CD8+ T cells. (f) Two months after immunization, serum levels of AMHR2-CD-specific IgG were detectable even at titers over 1 : 50,000 dilution. PBS was substituted for diluted sera in the PBS control. Error bars show ±SD.

Ten days after AMHR2-CD immunization of female C57BL/6 mice, LNC showed proliferation in a dose response manner to AMHR2-CD but not to recombinant human cochlin, a control protein generated and purified in a manner similar to AMHR2-CD (Figure 2(a)) [27]. This antigen-specific proliferation by LNC was elicited from purified CD4+ T cells but not from purified CD8+ T cells (Figure 2(b)) and was inhibited by treatment of cultures with CD4-specific but not CD8-specific antibodies (Figure 2(c)). Four weeks after immunization, ELISA analysis of supernatants from immunogen-stimulated splenocytes showed a predominant proinflammatory response to AMHR2-CD with high production of interferon gamma (IFN*γ*) and with relatively low production of IL-2, IL-4, and IL-5 (Figure 2(d)). Purification of T cell subsets from the whole splenocyte population showed that CD4+ but not CD8+ T cells produced the IFN*γ* in response to AMHR2-CD (Figure 2(e)). Two months after immunization, serum levels of AMHR2-CD-specific IgG were detectable even at titers exceeding 1 : 50,000 dilution (Figure 2(f)).

### 3.3. Benign Transient Ovarian Inflammation following AMHR2-CD Immunization

**Figure 3:**
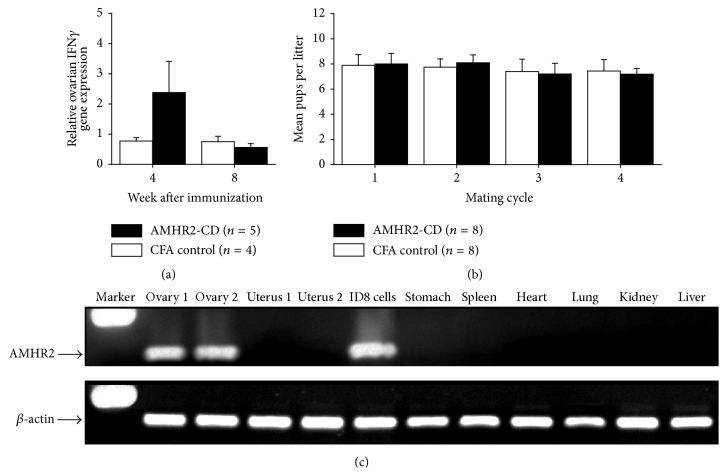
*Benign transient ovarian inflammation following AMHR2-CD immunization.* (a) Relative ovarian IFN*γ* gene expression was elevated 4 weeks after immunization with AMHR2-CD but not after immunization with CFA alone. At eight weeks after immunization, relative ovarian IFN*γ* gene expression was similar in both immunized groups of mice. (b) The low level transient expression of IFN*γ* in ovaries of AMHR2-CD immunized mice was not associated with any detectable effect on ovarian function as determined by assessing fertility defined by pup production over four sequential mating cycles in female C57BL/6 mice immunized with AMHR2-CD and control mice immunized with CFA alone. (c) AMHR2 gene expression was confined to ovaries and ID8 ovarian tumor cells and was not detected in normal uterus, stomach, spleen, heart, lung, kidney, and liver. Error bars show ±SD.

We next examined the potential of AMHR2-CD immunization to induce ovarian autoimmunity. Four and eight weeks after AMHR2-CD immunization of C57BL/6 female mice, ovarian IFN*γ* gene expression was measured by qRT-PCR. Relative ovarian IFN*γ* gene expression was modestly elevated 4 weeks after AMHR2-CD immunization but not after immunization with CFA alone (Figure 3(a)). Eight weeks after immunization, relative ovarian IFN*γ* gene expression was similar in both immunized groups of mice. Most notably, the transiently elevated IFN*γ* gene expression observed in AMHR2-CD immunized mice at 4 weeks was only 3-fold higher than CFA control mice, far lower than what we had previously observed in lactating breast tissues from mice immunized with *α*-lactalbumin where the levels of IFN*γ* gene expression were more than 50 times greater than those occurring in CFA immunized control mice and were associated with substantial breast inflammation and lactation failure [26]. Despite repeated attempts to detect CD3+ T cells in ovaries by immunohistochemical analysis at 4, 8, and 12 weeks after AMHR2-CD immunization, we could not find any infiltrates. More importantly, the low level transient expression of IFN*γ* in ovaries of AMHR2-CD immunized mice was not associated with any detectable effect on ovarian function determined by fertility over four sequential mating cycles during which no significant differences (*P* > 0.60) occurred in the number of pups generated per litter between AMHR2-CD and CFA immunized mice (Figure 3(b)). Moreover, it remains highly unlikely that AMHR2-CD immunization induces any substantial nonovarian autoimmune inflammation since we found that AMHR2 gene expression was readily detected in the ovaries and ID8 ovarian tumor cells and was not detected at any appreciable levels in normal mouse uterus, stomach, spleen, heart, lung, kidney, and liver (Figure 3(c)).

### 3.4. Inhibition of Tumor Growth in Mice Immunized with AMHR2-CD

**Figure 4:**
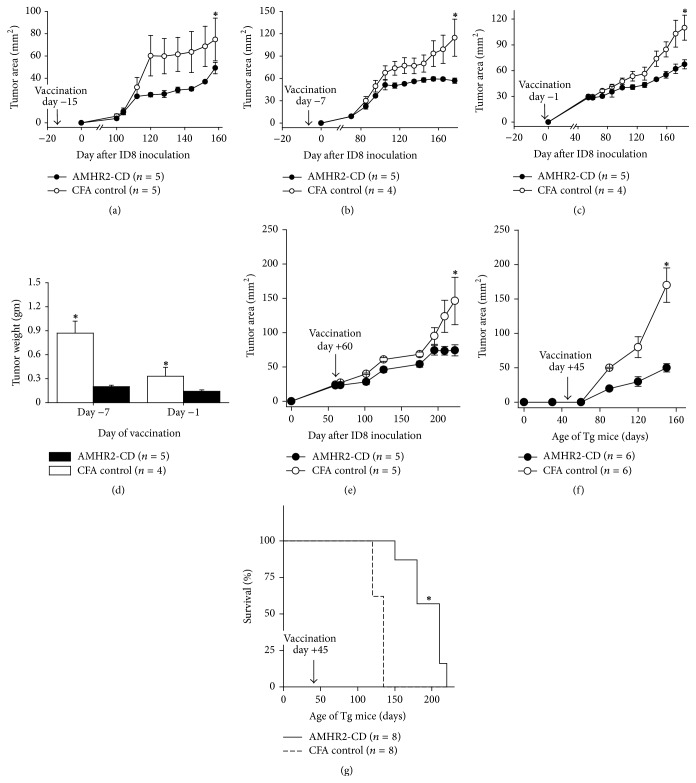
*Inhibition of tumor growth in mice immunized with AMHR2-CD*. ID8 tumor growth was inhibited in mice prophylactically vaccinated (a) 15 days, (b) 7 days, or (c) 1 day prior to inoculation of tumor cells. (d) AMHR2-CD vaccination resulted in a significantly decreased overall tumor load as measured by final tumor weight at termination of experiments in mice vaccinated 7 days and 1 day prior to ID8 inoculation. (e) Therapeutic vaccination with AMHR2-CD 60 days after inoculation of ID8 tumors significantly inhibited the growth of established, palpable, and growing ID8 tumors. (f) Prophylactic vaccination of female TgMlSIIR-TAg transgenic mice at 6-7 weeks of age with AMHR2-CD resulted in a highly significant inhibition in growth of autochthonous EOC. (g) Prophylactic AMHR2-CD vaccination of female TgMlSIIR-TAg transgenic mice at 6-7 weeks of age resulted in a highly significant 41.7% mean increased OS compared to control mice vaccinated with CFA alone. Asterisks indicate statistical significance. Error bars show ±SD.

We next determined whether vaccination with AMHR2-CD would inhibit growth of transplantable ID8 tumors in C57BL/6 female mice. We found that ID8 tumor growth was inhibited in mice prophylactically vaccinated 15 days (Figure 4(a),* P* < 0.001), 7 days (Figure 4(b),* P* < 0.001), or 1 day (Figure 4(c),* P* < 0.05) prior to inoculation of ID8 ovarian tumor cells. In addition, AMHR2-CD vaccination resulted in a significantly decreased overall tumor load as measured by final ID8 tumor weight at termination of experiments in mice vaccinated 7 days (*P *< 0.01) and 1 day (*P* < 0.05) prior to ID8 inoculation (Figure 4(d)). AMHR2-CD vaccination was also effective as therapy against EOC. Vaccination with AMHR2-CD 60 days after inoculation of ID8 tumors significantly inhibited the growth of established, palpable ID8 tumors (*P *< 0.05, Figure 4(e)). We also found that vaccination with AMHR2-CD significantly inhibited the growth of autochthonous EOCs that develop spontaneously in TgMlSIIR-TAg transgenic mice (*P* < 0.0001, Figure 4(f)). Moreover, this inhibition in tumor growth was accompanied by a highly significant increased OS when compared to CFA vaccinated control mice (*P* < 0.0005, Figure 4(g)). This enhanced lifespan in AMHR2-CD vaccinated mice (mean 191.25 days ±22.95) compared to CFA vaccinated control mice (mean 135 days ±13.89) represents a dramatic 41.7% increase in OS.

### 3.5. ID8 Tumor Analysis

**Figure 5:**
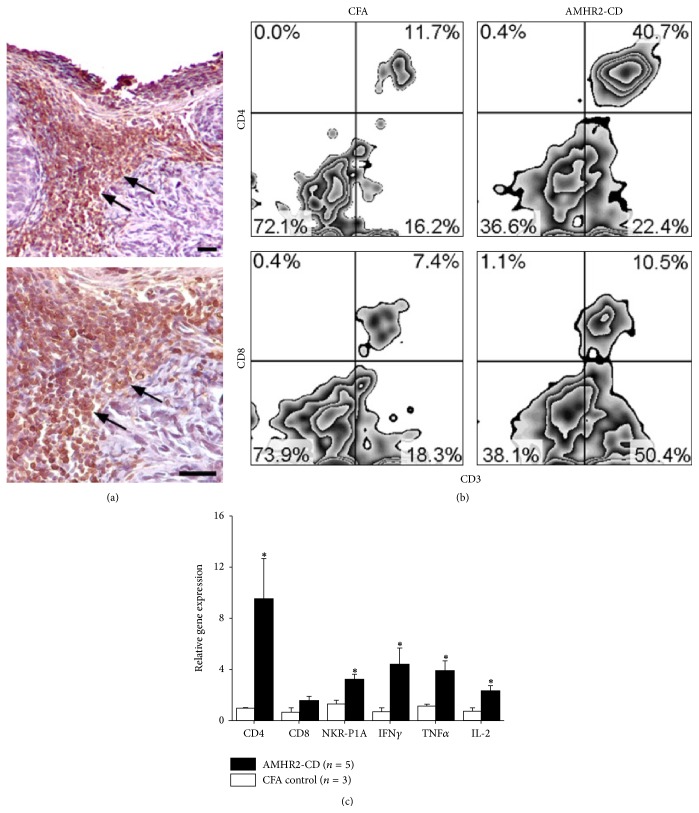
*Tumor analysis*. (a) Arrows show extensive infiltration of CD3+ T cells in an ID8 tumor from AMHR2-CD vaccinated mice in lower resolution (upper panel) and higher resolution (lower panel) images. Inflammatory infiltrates of CD3+ T cells were never observed in control mice vaccinated with CFA alone. (b) Flow cytometry analysis of TILs gated on the CD3+ T cell population showed a pronounced increase in percentages of CD4+ T cells but not CD8+ T cells in tumor infiltrates from mice vaccinated with AMHR2-CD compared to control mice immunized with CFA alone. Data shown are representative of three experiments yielding similar results. (c) Tumors from AMHR2-CD immunized mice consistently showed increased relative gene expression for CD4, IFN*γ*, TNF*α*, NKR-P1A, and IL-2 but not for CD8. Asterisks indicate statistical significance. Error bars show ±SD.

At the termination of experiments, tumors were analyzed for inflammatory infiltrates. Immunohistochemical analysis consistently showed extensive infiltration of CD3+ T cells in tumors from AMHR2-CD vaccinated mice (Figure 5(a)). We found no infiltrates in tumors from mice immunized with CFA alone (data not shown). Flow cytometry analysis of TILs showed a pronounced increase of CD4+ T cells in tumors from mice vaccinated with AMHR2-CD compared to control mice immunized with CFA alone (40.7% versus 11.7%, Figure 5(b)). Substantial increases of CD8+ T cells in tumors did not occur in AMHR2-CD immunized mice compared to CFA immunized control mice (10.5% versus 7.4%, resp.). We next analyzed tumor RNA for gene expression of proinflammatory factors by qRT-PCR. When compared to tumors from CFA immunized control mice, tumors from AMHR2-CD immunized mice consistently showed significantly increased relative gene expression (*P *< 0.05 in all cases) for CD4, IFN*γ*, tumor necrosis factor alpha (TNF*α*), IL-2, and the natural killer cell receptor NKR-P1A [28] but not for CD8 (Figure 5(c)). These data indicate the induction of a proinflammatory immune milieu within the ID8 tumor following immunization with AMHR2-CD.

### 3.6. Passive Transfer of Tumor Immunity with CD4+ T Cells

**Figure 6:**
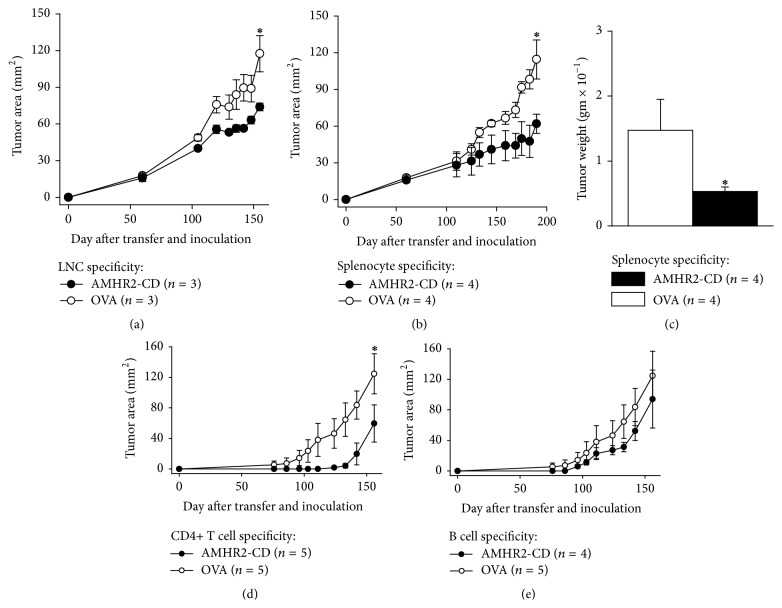
*Passive transfer of immune protection against tumor growth with CD4+ T cells*. Recipient mice were inoculated with ID8 tumor cells on the day after cell transfer. Growth of ID8 tumors was inhibited in mice transferred with AMHR2-CD-specific (a) LNCs and (b) splenocytes. (c) At 190 days after splenocyte transfer and inoculation, mean tumor weights were lower in recipients of AMHR2-CD-specific splenocytes compared to recipients of OVA-specific splenocytes. Transfer of purified AMHR2-CD-specific (d) CD4+ T cells but not (e) B220+ B cells inhibited ID8 tumor growth. Asterisks indicate statistical significance. Error bars show ±SE.

All recipient mice were inoculated with ID8 tumor cells on the day after cell transfer. Tumor growth was significantly inhibited in mice transferred with AMHR2-CD-specific LNCs (*P* = 0.04, Figure 6(a)) and splenocytes (*P* < 0.01, Figure 6(b)) when compared to mice receiving OVA-specific LNCs. At 190 days after transfer of primed splenocytes and tumor inoculation, mean tumor weights were significantly lower in recipients of AMHR2-CD-specific splenocytes compared to recipients of OVA-specific splenocytes (*P* < 0.05, Figure 6(c)). Transfer of AMHR2-CD-specific CD4+ T cells purified from 4-week primed splenocytes resulted in significant inhibition of ID8 tumor growth compared to transfer of purified OVA-specific CD4+ T cells (*P* < 0.0004, Figure 6(d)) whereas transfer of AMHR2-CD-primed B220+ B cells purified from 4-week primed splenocytes did not significantly inhibit ID8 tumor growth compared to transfer of OVA-primed B220+ B cells (*P* = 0.07, Figure 6(d)). Thus, AMHR2-CD-specific proinflammatory CD4+ T cells are sufficient for transferring immune protection against the growth of EOC.

## 4. Discussion

Our data derived from both transplantable and autochthonous ovarian tumor models show that vaccination against AMHR2-CD, a defined fragment of an ovarian differentiation protein expressed in the vast majority of human EOCs, provides effective therapy and prophylaxis against ovarian cancer. It is particularly encouraging that the inhibition of tumor growth was accompanied by a mild ovarian inflammation that resolved quickly with no detectable effects on fertility over the course of several subsequent mating cycles. This rather benign autoimmune phenotype was associated with a significant inhibition of tumor growth when vaccination occurred as a therapeutic intervention. It is important to note that the appearance of the therapeutic effect took over five months to clearly manifest as defined by a complete separation of the tumor growth curves (Figure 4(e)), thereby implying that earlier vaccination as a preventive strategy would be even more effective in controlling EOC. Indeed, the highly significant 41.7% increased OS that occurred when TgMISIIR-TAg mice were vaccinated prophylactically supports this view. However, in light of several reports indicating extraovarian gene and protein expression of AMHR2, the likelihood of using AMHR2-CD vaccination as prophylaxis against EOC seems unlikely.

*AMHR2* gene and protein expression have been repeatedly detected in adult motor neurons in mice [29–32], in normal adult rat endometrium, at low levels in the normal rat uterus, and at substantially higher levels in the gravid rat uterus [33]. Although* AMHR2* gene expression has also been shown to occur in brain, adrenal, and lung tissues of adult male mice, the detected levels were less than 1% of those occurring in adult testes, and protein detection was either not reported or not prominent [34]. Similarly,* AMHR2* gene expression has been detected in normal rat and normal human breast tissues, but detection of the AMHR2 protein was not reported in either of these tissues [35].

Despite extensive literature on extraovarian gene expression of* AMHR2*, recent rigorous studies have provided more precise understanding of the subtle but important features involved in* AMHR2* gene expression in different normal human tissues. Quantitative estimates of transcript abundance by mRNA sequencing have shown that expression of the full-length AMHR2 transcript is confined to the ovary in adult women whereas alternative splice isoforms coding for known truncated AMHR2 proteins as well as several noncoding transcripts are expressed in the cortex and medulla of the adrenal gland and at substantially lower levels in the spleen and exocrine cells of the pancreas [17]. Expression of truncated isoforms of AMHR2 also occurs in human skeletal muscle and heart but at levels, respectively, representing only 4.2% and 1.5% of the level expressed in the ovary. Thus, it seems that the extraovarian expression of AMHR2 reported in the literature refers to alternatively spliced transcripts that either code for truncated variant AMHR2 proteins or represent noncoding transcripts with no open reading frames [16, 17]. Noncoding AMHR2 transcripts have been shown to play a role in regulating AMHR2-mediated signaling [34] whereas all of the truncated AMHR2 transcripts with open reading frames have substantial deletions in the cytoplasmic domain of AMHR2 and as such are not capable of translating the complete AMHR2-CD sequence [16, 17]. Thus, the substantial AMHR2 deletions in nonovarian tissues may preclude the development of any life-threatening peripheral autoimmunity as evidenced by the lack of any observed extraovarian autoimmunity in our AMHR2-CD vaccinated mice and by the dramatically increased OS occurring in female TgMISIIR-TAg transgenic mice vaccinated prophylactically against AMHR2-CD.

The lack of any observed nonovarian autoimmunity in females provides several noteworthy considerations when selecting cancer vaccine targets including the importance of recognizing differences between immunogenic and nonimmunogenic tissue expression levels when anticipating autoimmune consequences of cancer vaccination. In addition, one must evaluate the significance of species-specific differences in tissue expression of cancer vaccine targets, since, unlike the mouse [35], AMHR2 transcripts have not been detected in any of the normal human brain tissues examined [17]. In any event, extraovarian expression of AMHR2 transcripts does little to diminish the usefulness of AMHR2-CD as an immune target for immunotherapy of EOC particularly in light of the urgent need to improve the poor prognosis of women diagnosed with EOC and the unusually high immunogenicity of AMHR2-CD indicated by T cell production of high levels of IFN*γ* and induction of extremely high serum antibody titers with prominent detection of AMHR2-CD-specific IgG occurring even at serum dilutions exceeding 1 : 50,000 (Figure 2(f)).

It is notable that a single immunization with AMHR2-CD is capable of inducing sufficient tumor immunity without eliciting a detectable CD8 T cell response. Although immunization with tissue-specific self-proteins often fails to elicit CD8 T cell responses, it may be presumptuous to conclude that such failure is due to the single immunization protocol. Indeed, we have previously shown that CD4 and CD8 T cell responses occur following single immunizations with *α*-lactalbumin and uroplakin II for effective induction of autoimmune breast failure and interstitial cystitis, respectively [26, 36]. In fact, booster immunizations often diminish effective immunity [37]. Thus, failure to induce CD8 T cell responses cannot be explained simply by inadequate priming and may instead be due to the unavailability of autoreactive CD8 T cells capable of responding to a specific self-protein possibly as a result of a more efficient thymic deletion of the high affinity CD8 T cell repertoire. In such cases, booster immunizations would simply recruit low affinity T cell clones representing a nondominant or cryptic T cell repertoire capable of limited clinical impact. Although optimal tumoricidal activity may typically occur when tumor responses involve both CD4 and CD8 T cells [38], CD4 T cells by themselves can provide powerful tumor immunity often exceeding that provided by CD8 T cells even when tumors fail to express major histocompatibility complex (MHC) class II molecules [39]. Studies to determine the basis for the unusually high immunogenicity of AMHR2-CD are ongoing.

A variety of mechanisms may contribute to CD4-mediated tumor immunity including induction of help for tumor responsive CD8+ T cell responses, induction of tumor cytotoxicity, upregulation of expression of MHC molecules for enhancing recognition of tumor antigens, inhibition of angiogenesis, and induction of tumor dormancy (reviewed in [40]). Most of these mechanisms are directly or indirectly related to upregulation of IFN*γ* and TNF*α* gene expression both of which occurred in tumors from mice vaccinated against AMHR2-CD (Figure 5(c)). Moreover, enhanced gene expression for the natural killer cell receptor, NKRP1A, in tumors from mice vaccinated against AMHR2-CD (Figure 5(c)), implies that NK cells may also play a role in the observed tumor immunity perhaps as a result of recruitment through IFN*γ*-dependent CXCR3 signaling [41] or through an IL-17/CCL2 recruitment mechanism [42]. Thus, CD4+ T cells may also mediate upregulated expression of angiostatic chemokines such as CXCL9, CXCL10, and CXCL14 that are capable of inhibiting tumor growth (reviewed in [40]) or may downregulate expression of the chemokine receptor CXCR2 or its many ligands that promote angiogenesis (reviewed in [43, 44]). Studies are currently underway to distinguish the underlying mechanism(s) involved in the therapeutic efficacy of AMHR2-CD vaccination against EOC.

## 5. Conclusion

In several mouse models of EOC, a single vaccination against AMHR2-CD is sufficient to provide effective immune control over the growth of ovarian tumors. Notably, this vaccine-induced tumor immunity occurs in the absence of any severe autoimmune consequences. Thus, AMHR2 vaccination may be useful in controlling the more malignant forms of human ovarian cancer.

## Figures and Tables

**Table 1:** Primer pairs used for cloning, qRT-PCR, detection of transgene, and conventional RT-PCR.

Protein	Sequence (5′-3′)	Amplicon length (bp)
*Cloning of AMHR2-CD*		
AMHR2-CD		
Forward	GGATCCAAGGCCTGCAGAGTGCAAGGTG	1209
Reverse	AAGCTTCTACTCATTTACATACACCTG	
*TgMISIIR-TAg transgene expression*		
SV40-TAg		
Forward	TGCATGGTGTACAACATTCC	773
Reverse	TTGGGACTGTGAATCAATGCC	
*qRT-PCR*		
IFN*γ*		
Forward	GGATATCTGGAGGAACTGGCAA	110
Reverse	TGATGGCCTGATTGTCTTTCAA	
TNF*α*		
Forward	CGAGTGACAAGCCTGTAGCC	209
Reverse	GTGGGTGAGGAGCACGTAGT	
IL-2		
Forward	GCAGGCCACAGAATTGAAAG	207
Reverse	TCCACCACAGTTGCTGACTC	
CD4		
Forward	ACACACCTGTGCAAGAAGCA	69
Reverse	GCTCTTGTTGGTTGGGAATC	
CD8		
Forward	TTACATCTGGGCACCCTTG	132
Reverse	TTGCCTTCCTGTCTGACTAGC	
NKR-P1A		
Forward	GGCTTGGCATGAGTCACC	75
Reverse	TTCAGAGCCAACCTGTGTGA	
*β*-actin		
Forward	GGTCATCACTATTGGCAACG	133
Reverse	ACGGATGTCAACGTCACACT	
*Conventional RT-PCR*		
AMHR2		
Forward	GTATCCGCTGCCTCTACAGC	193
Reverse	CAGAAGTCAGTGCCACAGGA	
*β*-actin		
Forward	GGTCATCACTATTGGCAACG	133
Reverse	ACGGATGTCAACGTCACACT	

## Abbreviations

AMH: Anti-Müllerian hormone
AMHR2: Anti-Müllerian hormone receptor 2
AMHR2-CD: Anti-Müllerian hormone receptor, type II-cytoplasmic domain
AIRE: Autoimmune regulator transcription factor
CA-125: Cancer antigen 25
CTAG1B or NY-ESO-1: Cancer-testis antigen 1
CFA: Complete Freund's adjuvant
cpm: Counts per minute
DC: Dendritic cell
EOC: Epithelial ovarian cancer
HPLC: High performance liquid chromatography
HRP: Horse radish peroxidase
HER2/neu: Human epidermal growth factor receptor tyrosine kinase
IFN*γ*: Interferon gamma
IPTG: Isopropyl *β*-D-1-thiogalactopyranoside
NKR-P1A: Natural killer cell lectin-like receptor subfamily B, member 1
LNC: Lymph node cells
MHC: Major histocompatibility complex
mTECs: Medullary thymic epithelial cells
MOSEC: Mouse ovarian surface epithelial cells
Ni-NTA: Nickel-nitrilotriacetic acid
NK: Natural killer
OS: Overall survival
OVA: Ovalbumin
qRT-PCR: Quantitative real-time RT-PCR
SV40-Tag: Simian virus 40 large T antigen
TCR: T cell receptor
TGF*β*: Transforming growth factor *β*
TILs: Tumor infiltrating lymphocytes
TAA: Tumor associated antigen
TNF*α*: Tumor necrosis factor alpha.
